# Effect of Bleomycin Hydrolase Gene Polymorphism on Late Pulmonary Complications of Treatment for Hodgkin Lymphoma

**DOI:** 10.1371/journal.pone.0157651

**Published:** 2016-06-21

**Authors:** Ádám Jóna, Zsófia Miltényi, Szilárd Póliska, Bálint László Bálint, Árpád Illés

**Affiliations:** 1 Department of Hematology, University of Debrecen, Faculty of Medicine, Debrecen, Hungary; 2 Genomic Medicine and Bioinformatic Core Facility, Department of Biochemistry and Molecular Biology, University of Debrecen, Faculty of Medicine, Debrecen, Hungary; University of Giessen Lung Center, GERMANY

## Abstract

**Background:**

Bleomycin hydrolase (BLMH), an enzyme that inactivates bleomycin, may be a potential candidate that could influence pulmonary function in ABVD (doxorubicin, bleomycin, vinblastin, dacarbasine)–treated Hodgkin lymphoma (HL) patients.

**Patients and Methods:**

We hypothesized that the BLMH gene SNP A1450G (rs1050565) influences BLMH activity and late pulmonary toxicity. St. George Respiratory Questionnaire, lung scintigraphy and spirometry were used to determine lung function. TaqMan genotyping assay was used to determine genotype distribution of 131 previously treated HL patients.

**Results:**

Significantly more favorable results were seen in the wild-type A/A genotype group than those in the group containing the mutated allele: A/G+G/G in retrospective pulmonary tests of ABVD treated patients.

**Conclusion:**

Besides limitations of the current study, bleomycin pharmacokinetics should be further evaluated in patients with BLMH variations, hence identify those cases even in the frontline setting, where bleomycin should be omitted and replaced with targeted therapy.

## Introduction

The survival rates of Hodgkin lymphoma (HL) patients have significantly improved over the past decades. Hence, reducing late toxicity along with better survival is of interest. Currently used standard of care, first line therapy, bleomycin-containing ABVD (doxorubicin, bleomycin, vinblastin, dacarbasin) and BEACOPP (bleomycin, etoposide, doxorubicin, cyclophosphamide, vincristine, procarbazine, prednisone) have been reported to produce bleomycin- induced lung injury in 20–46% of patients. [1,2]

Bleomycin, a glycopeptides antibiotic, was discovered in 1966, derived from *Streptomyces verticillus*, and later came to be used as an antitumor agent due to its capacity to induce reactive oxygen radicals. [3] Bleomycin is mainly excreted by the kidneys; however, it can also be eliminated by its specific deactivating enzyme, bleomycin hydrolase (BLMH). [4] Despite being an effective chemotherapeutic agent, its use is limited by its potentially life-threatening pulmonary toxicity. [5] Its toxic effect is related to its free radical-producing capacity, while its lung specificity is related to the fact that BLMH is absent in the lungs. [2] Risk factors for lung damage are as follows: cumulative bleomycin dose, chest irradiation, age, renal function, smoking, use of colony stimulating factors, and route of administration (intravenous (iv.) or intramuscular (im.)).

Bleomycin hydrolase (BLMH), an enzyme that inactivates bleomycin, may be a potential candidate that influences pulmonary function. Single nucleotide polymorphisms (SNP) A1450G is located in the C-terminal region, which is responsible for the enzymatic activity [6,7], and this SNP leads to the substituition of isoleucin as amino acid residue 443 by valine, thus changing aminopeptidase activity. [8] Although the exact effect of this SNP on the enzymatic activity of BLMH is unknown, it appears to influence the level of bleomycin-induced DNA damage. [9] No similar data about BLMH is available either in HL tissue *in vitro*, or among HL patients. [2]

We hypothesize that BLMH gene SNP A1450G influences BLMH activity and by an altered metabolism of bleomycin, differences can be seen at the risk of bleomycin-induced toxicity appearance. This study aims to evaluate treatment-related long-term pulmonary side effects and their correlation with genetic distribution.

## Patients and Methods

The current pulmonary function of HL patients previously treated at the Department of Hematology, University of Debrecen (UD), was investigated over a one-year-period as previously reported. [10] Briefly, after collecting epidemiological/ clinicopathological data (gender, age at time of diagnosis, clinical stage, histological subtypes, smoking history, pretreatment kidney function, treatment modality, chemotherapy regimen, radiotherapy modality and elapsed time from treatment), pulmonary function assessed using the St. George Respiratory Questionnaire (SGRQ), dynamic inhalation lung scintigraphy and spirometry, retrospectively at the outpatients clinic during their follow- up at a certain time point. SGRQ scores range between 0 and 100. The higher the value, the worse patient-reported quality of life. Spirometry results were calculated as a percentage of healthy, age and gender matched controls. To investigate possible functional deviations dynamic inhalation lung scintigraphy was performed. After inhalation, clearance of technetium-99m diethylene triamine penta-acetic acid (DTPA) was compared to the normal values of the corresponding age and lung side. The lower the result, the more severe the membrane lesion was.

After approval of the “Local Research Ethics Committee, Faculty of Medicine, University of Debrecen” and written informed consent of the patients, already surveyed, peripheral blood samples were collected of previously treated HL patients, whose current pulmonary function test results were already available. Patient samples were blinded by receiving unique codes, therefore during identifying their genotype authors weren’t aware pf patients’ identity. Genomic DNA was isolated from peripheral blood samples using MagnaPure 96 System (Roche) according to manufacturer’s protocol. SNP A1450G (rs1050565) of the BLMH gene was genotyped using TaqMan genotyping assays (Lifetechnologies). Measurements and genotype calling were performed on QuantStudio 12K flex instrument (Lifetechnologies) at the Genomic Medicine and Bioinformatic Core Facility of UD. The QC of the genotyping calls were >98% and the technical duplicates gave the same genotype in every cases.

The genotype results of BLMH were then compared with current pulmonary status, to conclude, whether BLMH SNP A1450G correlates with long-term pulmonary toxicity.

Statistical analysis was performed using SPPS version 22 software. Analysis was done using the Mann-Whitney test, Chi-square test and linear regression analysis as appropriate. Correlations were considered significant if p<0.05.

## Results

Out of the total cohort of 131 previously treated HL patients, bleomycin was included in the treatment of 102 patients (ABVD, BEACOPP, COPP/ABV (cyclophophamide, vincristin, prednisone, procarbazine, doxorubicin, bleomycin, vinblastin), relapsed/ refractory patients). The treatment of the remaining 29 patients excluded bleomycin (irradiation only, COPP (cyclophophamide, vincristin, prednisone, procarbazine), CVPP (cyclophophamide, vinblastine, prednisone, procarbazine)), thus representing a control group. Out of the 102 bleomycin-treated patients 68 received ABVD chemotherapy alone. Median bleomycin dose and time elapsed from treatment completion differed significantly in the treatment groups. Other factors (smoking, age, bleomycin dose, chest irradiation, kidney function, use of colony stimulating factors) that could potentially affect lung function were equally represented in all investigated subgroups with no significant differences. Further patient characteristics are detailed in Table 1.

**Table 1:** Patient characteristics of Hodgkin lymphoma patients.

	Control (no bleomycin)	All bleomycin treated patients	p-value*	Only ABVD treated patients	p-value**
**Number of patients**	29	102		68	
**Bleomycin dose, mg/m2 (median, range)**	0	130 (40–160)	N/A	120 (60–160)	N/A
			N/A		
**Age, years (median, range)**	28 (14–57)	30 (14–73)	0.871	30 (15–62)	0.143
**Male/female**	16/13	55/47	0.764	40/28	0.740
**Years since treatment (median, range)**	28 (15–44)	10 (2–25)	<0.0001	8 (2–22)	<0.0001
***Number of patients (%)***					
**Stage**					
** early**	16 (55.17)	52 (51.00)	0.361	43 (63.24)	0.880
** advanced**	13 (44.83)	50 (49.00)		25 (36.76)	
**Histology**					
** NPLHL**	2 (6.90)	3 (2.94)	0.318	3 (4.41)	0.575
** cHL**	27 (93.10)	99 (97.06)		65 (95.59)	
**BLMH genotype**					
** G/G**	5 (17.24)	10 (9.80)		6 (8.82)	
** A/G**	10 (34.48)	34 (33.33)	0.296	22 (32.35)	0.253
** A/A**	14 (48.28)	58 (58.86)		40 (58.82)	
**Smokers**	5 (17.24)	29 (28.43)	0.645	22 (32.35)	0.673
**Chest irradiation**	13 (44.83)	49 (48.04)	0.727	31 (45.49)	0.945
**Use of G-CSF**	N/A	18 (17.65)	N/A	13 (19.12)	N/A
**Creatinine clearance, μmol/min (median, range)**	N/A	72 (47–99)	N/A	69 (45–98)	N/A

* Comparison of "All bleomycin treated patients" and "Controll group"

** Comparison of "Only ABVD treated patients" and "Controll group

A total of 131 patients were treated with either bleomycin-containing therapy (ABVD, BEACOPP, COPP/ABV, relapsed/ refractory patients) (n = 102) or excluding bleomycin, regarded as a control group (irradiation only, COPP, CVPP) (n = 29). Within the bleomycin-treated cohort a total number of 68 patients were treated with ABVD regimen alone. *p* values are considered significant if <0.05 and are marked bold. N/A—not applicable, ABVD—adriamycin (doxorubicin), bleomycin, vinblastin, dacarbazine, BEACOPP—bleomycin, etoposide, adriamycin (doxorubicin), cyclophosphamide, oncovin (vincristine), procarbazine, prednisone), COPP/ABV—cyclophophamide, vincristin, prednisone, procarbazine doxorubicin, bleomycin, vinblastin, COPP—cyclophophamide, vincristin, prednisone, procarbazine, CVPP—cyclophophamide, vinblastine, prednisone, procarbazine, NLPHL—nodular lmyphocyte predominat Hodgkin lymphoma, cLR—classical lymphocyte rich, cMC—classical mixed cellularity, cNS—classical nodular sclerosis, cLD—classical lymphocyte depletion, ND—not determined, G-CSF—granulocyte colony stimulating factor.

Patient samples were collected from these previously treated HL patients, whose BLMH A1450G genotype distribution was determined. Homozygous wild-type A/A genotype was found in 72 patients (55.0%), while 44 patients (33.6%) had heterozygous A/G and 15 patients (11.5%) had homozygous mutated G/G genotype, where “A” is the wild-type (allele frequency: 71.8%) and “G” is the mutated allele (allele frequency: 28.2%). (Table 2) Our results were comparable to the NCBI SNP database for BLMH SNP A1450G. (Table 3) Allele frequencies were in Hardy-Weinberg equilibrium. G/G genotype group alone would have been too small for relevant statistical analysis, therefore patients were then subdivided into subgroups: one containing the mutated allele: A/G+G/G (45.1%) and the other homogenous for the wild-type allele: A/A (55.0%), thus demonstrating the possible role of the wild-type “A” and mutated “G” allele.

**Table 2:** Allele and genotype frequency of Hodgkin lymphoma patients’. Own patients’ data (n = 131).

**Allele frequency**			
	A		G
**Pts. nr.**	188		74
**%**	71.8		28.2
**Genotype frequency**			
	A/A	A/G	G/G
**Pts. nr.**	72	44	15
**%**	55.0	33.6	11.5

“A” is the wild-type and “G” is the mutated allele. Patients were subdivided into subgroups: one containing the mutated allele: A/G+G/G (45.1%) and the other homogenous for the wild-type allele: A/A (55.0%). Pts. nr.–Patients number

**Table 3:** NCBI SNP database (rs1050565).

**Allele frequency**			
	A		G
**%**	71.8		28.2
**Genotype frequency**			
	A/A	A/G	G/G
%	41.7	45.8	12.5

“A” is the wild-type and “G” is the mutated allele. Our results are comparable with the NCBI database. NCBI—National Center for Biotechnology Information, SNP—Single nucleotide polymorphysm.

Factors, which could potentially affect lung function, were equally represented between the subgroups containing the mutated allele (A/G+G/G) and those homogenous for the wild-type allele (A/A) even within the investigated treatment groups with no significant differences. (Table 4)

**Table 4:** Factors that could potentially affect lung function were equally represented in each treatment subgroup.

	All bleomycin treated patients			Only ABVD treated patients			Control (no bleomycin)		
	A/G+G/G	A/A	*p*	A/G+G/G	A/A	*p*	A/G+G/G	A/A	*p*
**Number of patients**	43	59		28	40		15	14	
**Bleomycin dose, mg/m2 (median, range)**	140 (40–160)	130 (40–160)	0.440	140 (80–160)	130(60–160)	0.116	0	0	N/A
**Median age, years (range)**	44 (16–73)	33 (14–62)	0.967	44 (19–58)	33 (15–62)	0.653	26 (14–57)	24 (14–45)	0.756
**Male/female**	21/22	34/25	0.424	17/11	23/17	0.792	5/9	10/4	0.158
**Years since treatment (median, range)**	9 (2–25)	7 (1–25)	0.191	9 (2–16)	7 (1–22)	0.544	30 (14–41)	35 (14–44)	0.401
**Smokers**	13 (30%)	16 (27%)	0.350	10 (36%)	12 (30%)	0.403	2 (13%)	3 (21%)	0.792
**Chest irradiation**	24 (56%)	25 (42%)	0.209	14 (50%)	17 (43%)	0.544	7 (47%)	6 (43%)	0.880
**Creatinine clearance, μmol/min (median, range)**	78 (47–92)	72 (48–99)	0.684	77 (45–86)	69 (48–98)	0.776	N/A	N/A	N/A
**Use of G-CSF**	11 (26%)	6 (10%)	0.091	7 (25%)	6 (15%)	0.241	N/A	N/A	N/A

“A”–wild-type, ABVD—adriamycin (doxorubicin), bleomycin, vinblastine, dacarbazine, “G”–mutant type, G-CSF—granulocyte-colony stimulating factor

All bleomycin- treated patients (n = 102) had more favorable lung function test results in the A/A genotype group, with every investigated test method with significant differences in the forced vital capacity results (FVC), p = 0.006. (data not shown)

**Fig 1:**
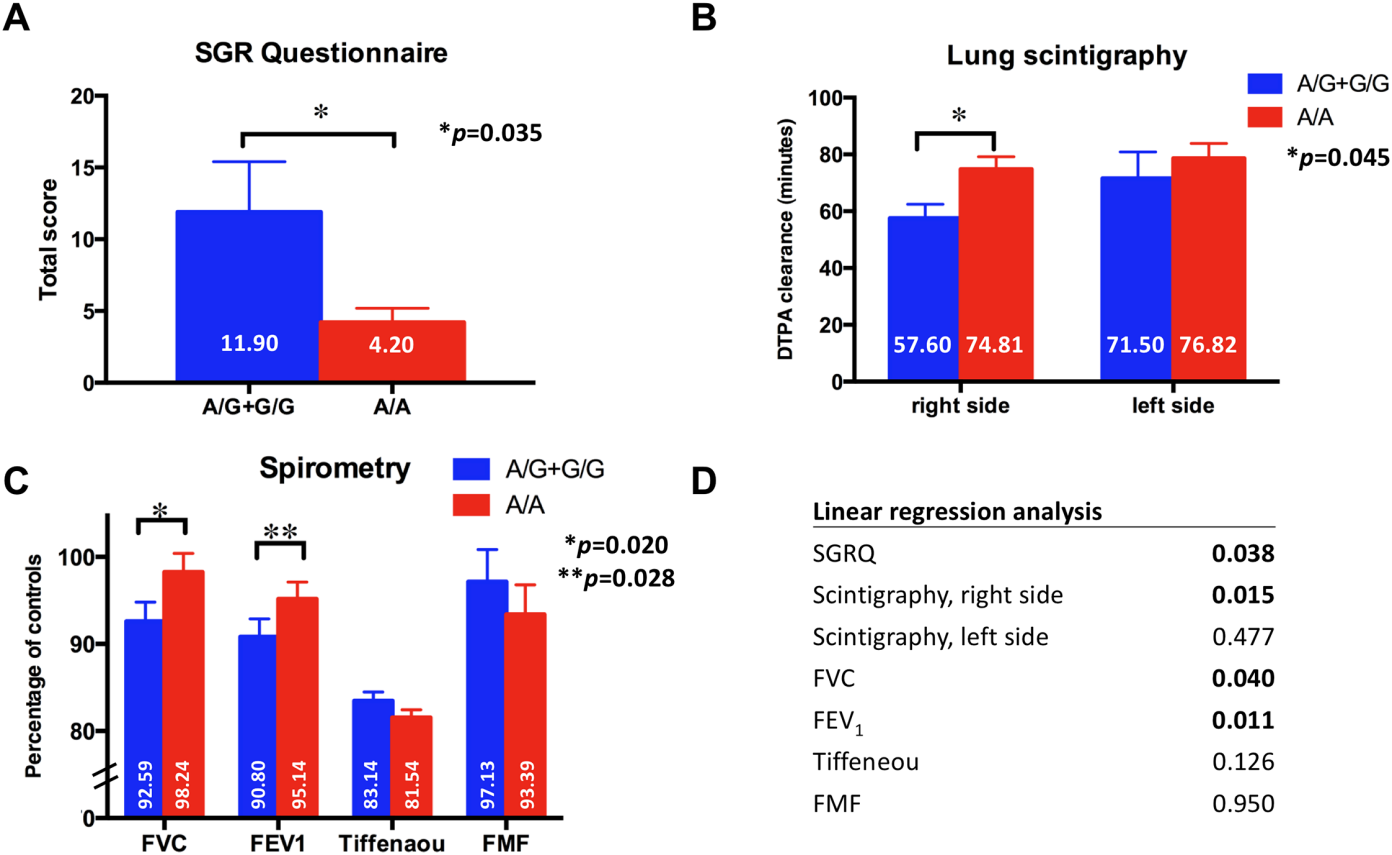
ABVD treated patients. A total number of 68 patients were treated with ABVD regimen alone. Mean value of subgroup ± standard error of mean (SEM) is shown. *p* values are considered significant if <0.05 and are marked bold. Significantly more favorable results were seen in the wild-type A/A genotype group of BLMH SNP A1450G with the St. George Respiratory Questionnaire (SGRQ) (**A.**), with lung scintigraphy results on the right side (**B.**). Spirometry revealed significantly more favorable forced vital capacity (FVC) and forced expiratory volume in 1 s (FEV1) results. (**C.**) Linear regression analysis of BLMH SNP A1450G confirmed these results for the different test methods. (**D.**) “A”–wild-type, ABVD—adriamycin (doxorubicin), bleomycin, vinblastine, dacarbazine, DTPA—diethylenetriamine penta-acetic acid, FEV_1_ –forced expiratory volume in 1 second, FMF—forced mid-expiratory flow, FVC—forced vital capacity, “G”–mutant type, SGRQ—St. George’s Respiratory Questionnaire

When focusing on patients who received ABVD regimen (n = 68) alone, significantly more favorable results were seen in the A/A genotype group, with every investigated test method. The SGRQ score was significantly more favorable in the A/A genotype group (11.90 pts. vs. 4.20 pts., p = 0.035). Right-sided lung scintigraphy results were also significantly more favorable in the A/A genotype group (74.81 vs. 57.56, p = 0.045). Among spirometry results FVC (p = 0.020) and forced expiratory volume in 1 s (FEV1) results were significantly more favorable (p = 0.028) in the A/A genotype group. A linear regression analysis also confirmed these results. (Fig 1)

**Fig 2:**
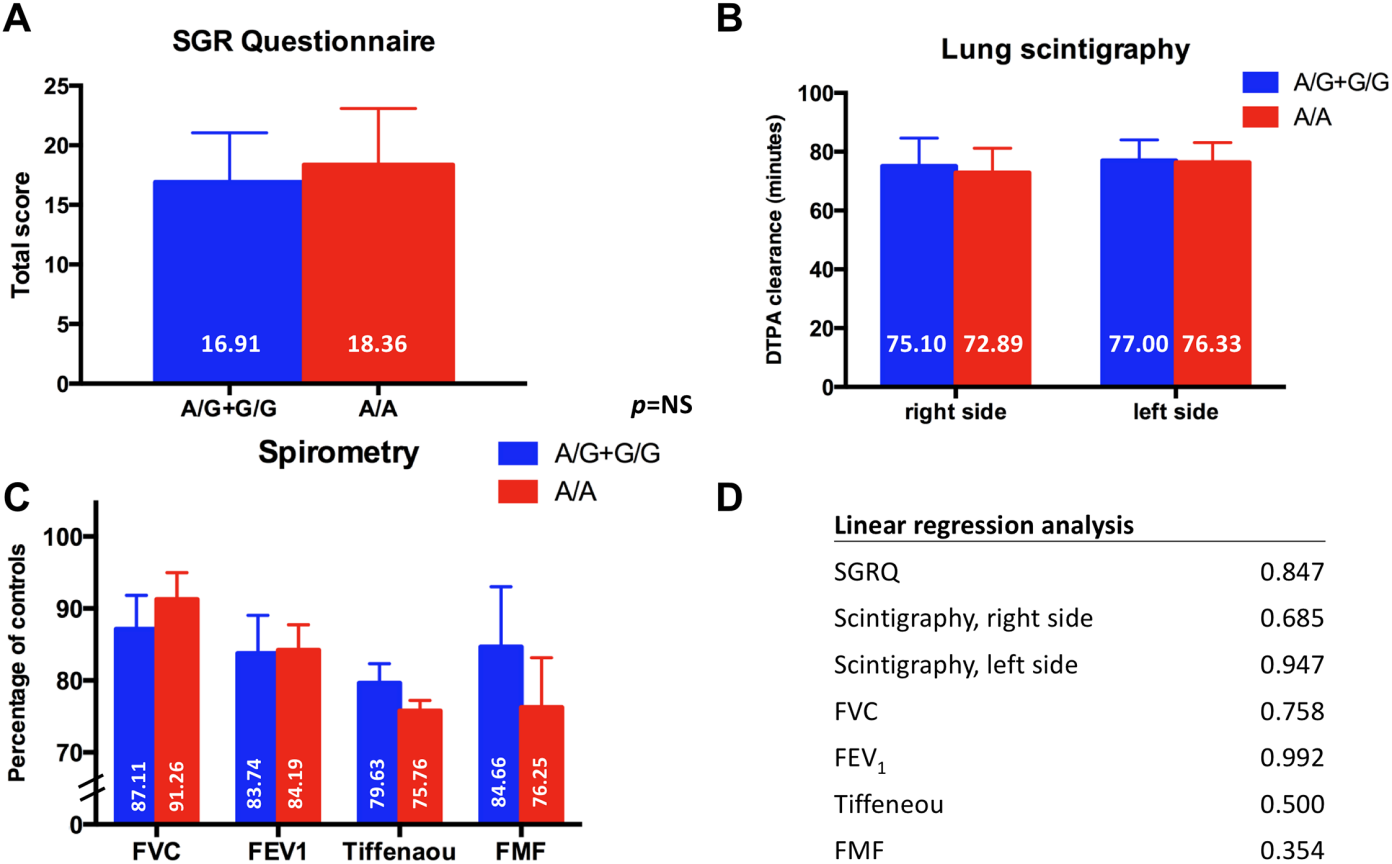
Control group. The control group contained 29 patients, whose treatment excluded bleomycin. Mean value of subgroup ± standard error of mean (SEM) is shown. No significant differences were seen between the A/G+G/G and A/A genotype groups. “A”–wild-type, DTPA—diethylenetriamine penta-acetic acid, FEV_1_ –forced expiratory volume in 1 second, FMF—forced mid-expiratory flow, FVC—forced vital capacity, “G”–mutant type; NS—non-significant, SGRQ—St. George’s Respiratory Questionnaire.

As a control group (n = 29), patients treated with agents excluding bleomycin (irradiation or chemotherapeutic regimens not containing bleomycin) were tested with no significant differences between A/G+G/G and A/A genotype groups. (Fig 2)

## Discussion

Bleomycin-induced lung injury is a valid limitation of current standard of care in HL patients. BLMH gene SNP A1450G polymorphism led to significant differences in the follow-up pulmonary test results of ABVD-treated HL patients. The authors believe, that the observed difference in the ABVD only group is due to the genotype differences, and not due to other factors, that could possibly affect pulmonary function, since these factors were equally represented between the treatment groups and also in the control group.

Bleomycin can be administered 10mg/m^2^ dose iv. or im.. In our institution patients are treated exclusively im. and dose is maximized at 15 mg. Given a median 120 mg/m2 bleomycin dose, a maximum of 180 mg bleomycin could have been administered. Only one acute bleomycin- induced lung injury was documented among the investigated patients. While the half-life of bleomycin is comparable with iv. and im. administration (2–3 hours), peak plasma level of im. administration is only one tenth of iv. administration, thus providing basis of barely lack of acute pulmonary toxicity. [11]

BLMH gene SNP A1450G has been investigated previously in testicular germ-cell cancer (TC) patients treated with bleomycin in correlation with survival data. Mutant variant (G/G) genotype was associated with decreased survival compared to the heterozygous (A/G) and the wild-type variants (A/A) [12]. SNP A1450G was also investigated in correlation with pulmonary toxicity of TC patients in a subsequent study. [13] Interestingly, no association was found of gene polymorphism either with acute bleomycin-induced pneumonitis or with pulmonary function tests performed during treatment. Because the serum half-life of bleomycin is relatively short, authors explained the lack of correlation with barely injured kidney function, when the effect of altered BLMH activity is negated by the rapid elimination of bleomycin. The fact, that homozygous variant (G/G) TC patients in the Dutch-study were associated with decreased survival could be explained by the fact, that in this genotype group, there were more patients with refractory disease, than in other groups. This suggest, that it may not have been an independent parameter. They describe, that G/G genotype variant could have been associated with other factors, that causes tumor resistance. The fact that they didn’t find association of SNPA1450G and acute pulmonary toxicity can be explained also, that they investigated the occurrence of acute pulmonary toxicity, whereas, our study investigated quantitative features of long-term pulmonary toxicity. Compared to the TC patients of the Dutch study, our patients were treated with lower cumulative bleomycin doses administered im., and still had normal kidney function results, possibly showing a major role of BLMH genetic polymorphism in inactivating bleomycin. Overall, BLMH gene SNP A1450G polymorphism seems to have significance on the long-term. Obviously, the different nature of the diseases may also have contributed to these conflicting results. Limitation of the control group is, that control patients cannot be matched in elapsed time since treatment with the investigated patient population. This is because we wanted to show; that BLMH had no role in those previously treated HL patients, who have not received bleomycin, however such treatment modality existed only formerly. The authors are aware, that this analysis has several limitations. We don’t have pharmacokinetic data available in the different genotype cases. Obviously this was a smaller cohort of patients, therefore replication and validation of our results needed. Replication in a bigger cohort would result in a situation, when we don’ t need to collapse heterozygous and homozygous variant carriers. Because bleomycin serum level is significantly affected by kidney function [14], evaluating bleomycin bioavailability in HL patients sera and demonstrating pharmacokinetics of bleomycin in patients with BLMH variations would confirm our results and correlation with BLMH gene SNP A1450G. Although this current analysis is only discovery-phase, an eventual goal would be to identify those cases with multiple pulmonary risk factors even in the frontline setting when brentuximab vedotin should be administered instead of bleomycin, either in standard of care or because of cost of targeted therapy. Eventually, treatment could be adjusted individually.

## Supporting Information

S1 File Results were calculated based on the “HL patients” dataset, while patient characteristics were evaluated based on “Clinicopathological features” data set. (XLS)
